# Dr. James McGarry

**Published:** 1903-09

**Authors:** 


Dr. James McGarry, of Niagara Falls, Ont., died at his home, August 13, 1903, aged 69 years. He was one of the best known physicians in his region, and was a citizen of prominence.

